# Postpartum Maternal Hypothermia in an Adolescent Patient Following Spontaneous Vaginal Delivery

**DOI:** 10.7759/cureus.28142

**Published:** 2022-08-18

**Authors:** Kara Weishaar, Erin Bailey, Hana El Ado Mikdachi, Jessica E Murphy

**Affiliations:** 1 Obstetrics and Gynecology, East Tennessee State University - Quillen College of Medicine, Johnson City, USA; 2 Obstetrics and Gynecology, Ascension Sacred Heart Women’s Care Center, Pensacola, USA; 3 Surgery/Gynecology, Jerry L Pettis VA Loma Linda Medical Center, Loma Linda, USA

**Keywords:** physiologic vasodilation of pregnancy, hypothermia, hypophyseal malfunction, postpartum complications, adolescent pregnancy, spontaneous vaginal delivery, postpartum hypothermia

## Abstract

Postpartum hypothermia, though rare after spontaneous vaginal delivery, can be life-threatening, warranting efficient workup and intervention. A 14-year-old primigravida developed postpartum hypothermia following spontaneous vaginal delivery. No clear etiology was identified despite extensive workup. Intervention with warmed fluids and application of forced air warming system resolved the hypothermia in less than 24 hours without relapse. Following negative workup, the most likely etiology was administration of chilled intravenous fluids in the setting of acute blood loss of delivery and physiologic vasodilation of pregnancy. This case demonstrates the importance of considering common and unusual causes of postpartum hypothermia and leads to a recommendation for routine postpartum temperature checks and hypothermia protocols that include warmed fluid replacement and a forced air warming system.

## Introduction

Peripartum hypothermia can be life-threatening, with significant maternal and neonatal consequences, thereby warranting efficient workup and intervention. Body temperature homeostasis is reliant on the balance between hypothalamic thermal regulation and external heat loss forces. Due to physiologic changes associated with pregnancy and parturition in addition to risk factors related to delivery, obstetric patients are particularly sensitive to temperature insult. Hypothermia is a well-recognized surgical complication that affects up to 50-91% of cesarean deliveries, however, hypothermia associated with vaginal delivery is extremely rare. We present a case of an adolescent patient who developed hypothermia following a precipitous vaginal delivery [1-3].

## Case presentation

A 14-year-old primigravida presented to the Pediatric Emergency Department for acute abdominal pain and was diagnosed with preterm labor at 34-35 weeks estimated gestational age by last menstrual period. Pelvic exam revealed cervical dilation of 10 cm, 100% effacement, and fetal station of +2 with membranes intact and bulging. The patient was emergently transferred to the obstetrics unit and precipitously delivered.

Her pregnancy was complicated by lack of prenatal care due to attempted concealment of pregnancy, history of transient heart murmur in early childhood, asthma, and unknown group B streptococcal colonization status. After delivery, she required repair of a left labial laceration and was given 5 mL lidocaine with 1% epinephrine for local analgesia. Her total estimated blood loss for delivery and subsequent repair was 150 mL. Upon admission, a complete blood count (CBC) and basic metabolic panel (BMP) were collected and resulted in the early postpartum period. Her CBC was within normal limits and BMP showed hypokalemia with mild hyponatremia and hypochloremia (Table 1). Intravenous (IV) normal saline with potassium chloride (KCl) 40 milliequivalent repletion was administered.

**Table 1:** Summary of Laboratory Evaluation and Results Pre-Delivery and Postpartum WBC: white blood cell, CO2: bicarbonate, BUN: blood urea nitrogen, AST: aspartate transaminase, ALT: alanine transaminase, TSH: thyroid stimulating hormone

Study	Admission Results	Postpartum Results			
		at 6 hours	at 23 hours	at 30 hours	
WBC	10.6	9.9	11	8.6	x10^3^/microL
Hemoglobin	11.4	11.6	10.9	10	g/dL
Hematocrit	33	33.3	32.1	28.6	%
Platelets	180	192	243	219	x10^3^/microL
Sodium	131	137	139	-	mEq/L
Potassium	2.6	3.4	3.3	-	mEq/L
Chloride	96	106	106	-	mEq/L
CO2	20	22	23	-	mEq/L
BUN	3	5	6	-	mg/dL
Creatinine	0.44	0.41	0.42	-	mg/dL
Calcium	8.8	8.6	8.5	-	mg/dL
Glucose	89	137	94	-	mg/dL
Magnesium	1.6	-	1.7	-	mg/dL
AST	-	-	13	-	IU/L
ALT	-	-	7	-	IU/L
Lactate	-	1.0	1.3	-	mmol/L
Procalcitonin	-	-	<0.20	-	ng/mL
Troponin	-	0.06	<0.03	<0.03	ng/mL
Cortisol	-	-	17	-	ug/dL
TSH	-	-	2.67	-	uIU/mL
Free T4	-	-	1.00	-	ng/dL
pH arterial	-	7.46	-	-	
pCO2 arterial	-	31	-	-	mmHg
pO2 arterial	-	98	-	-	mmHg
Base Excess	-	-0.9	-	-	mmol/L
pH venous	-	7.46	-	-	
pCO2 venous	-	34	-	-	mmHg
pO2 venous	-	42	-	-	mmHg
Base Excess	-	0.8	-	-	mmol/L

Her postpartum course was unremarkable until eight hours postpartum, when her body temperature was noted to be 92.5°F (33.6 C) orally by nursing staff, confirmed by rectal thermometer. Her blood pressure was 91/53 mmHg, her pulse was 64 bpm, and respiratory rate and oxygen saturation were within normal limits. No obvious etiology of her hypothermia was immediately evident.

**Figure 1:**
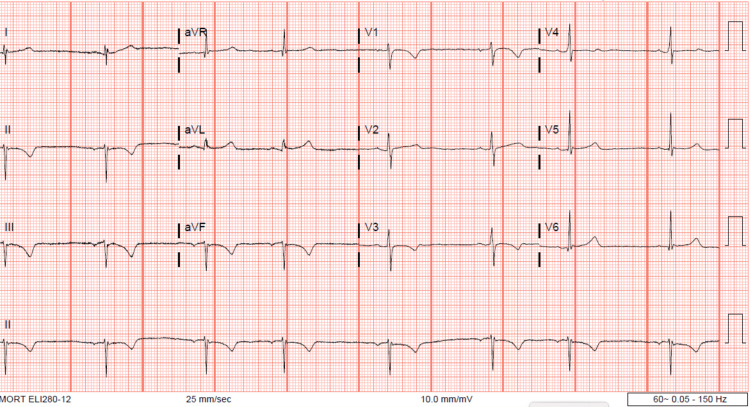
Postpartum EKG obtained during hypothermic episode demonstrating bradycardia. Pulse: 45

**Figure 2:**
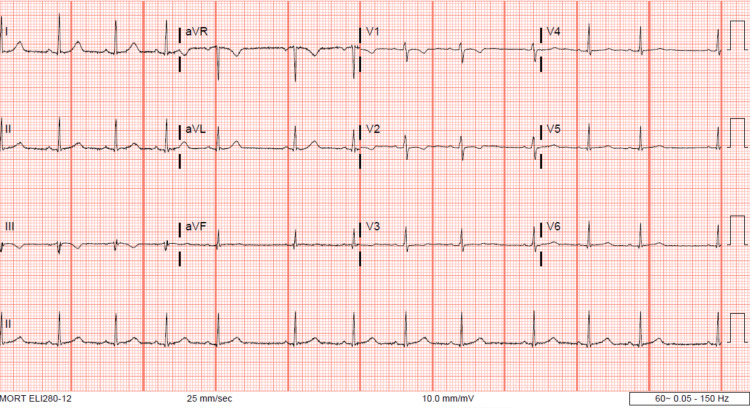
Admission EKG demonstrating normal sinus rhythm prior to delivery. Pulse: 74

The patient was given a 1-liter bolus of warmed Lactated Ringers solution and was placed in a forced air warming system. CBC, BMP, and serum lactate were collected. One hour later, a repeat rectal temperature was 93.1°F (33.9 C). Her vitals were otherwise stable, notable for bradycardia with a pulse of 45 bpm. Her laboratory studies did not show any acute abnormality. Her CBC was appropriate for expected postpartum changes. Her potassium level improved, with the normalization of other electrolytes, and her lactate level was within normal limits (Table 1). An electrocardiogram (EKG) confirmed ectopic atrial bradycardia (Figure 1). Figure 2 shows her baseline EKG obtained in the Emergency Department prior to transfer. The pediatric intensivist was consulted. Urinalysis, urine drug screen, arterial blood gases, blood cultures, and cardiac enzymes were ordered and empiric antibiotics (1g ceftriaxone) were administered. All studies resulted within normal limits apart from the urinalysis, which was contaminated.

The patient was admitted to the pediatric intensive care unit where supportive measures were continued and her temperature normalized. Additional workup included thyroid studies and serum cortisol, which were normal. After three days of evaluation, no source of her hypothermia was identified and the patient was discharged to home in stable condition.

The neonate’s hospital course was relatively uncomplicated apart from expected management of prematurity. Rupture of membranes occurred at the time of delivery within the hospital, a single nuchal cord was noted, and Apgar scores were 7 and 8 at 1 and 5 minutes, respectively. The Neonatal Intensive Care Unit (NICU) team were present for the delivery and the infant was admitted to the NICU for prematurity with continuous positive airway pressure (CPAP) support. Newborn vital signs were within normal limits except for a critically low blood pressure of 52/19. The initial point of care glucose was also critically low at 25 mg/dL. Arterial blood gases on CPAP were within normal limits. The CBC was also within normal limits. Blood cultures were collected and returned with no growth at five days.

Routine vaccinations were administered. Some initial difficulty with temperature regulation, consistent with prematurity, was documented. Over the next several days of life, the temperature remained stable under radiant warmer, glucose and blood pressure improved, and CPAP was weaned to room air. Phototherapy was administered for elevated bilirubin. The infant was tolerating full oral feeds and self-regulating temperature by Day of Life 15 and was discharged to home.

## Discussion

Hypothermia is defined by a core body temperature below 35°C (95°F), caused by excessive cold stress with inadequate thermogenesis. Body temperature is regulated by the hypothalamus and maintained through the autonomic nervous system using input from thermal receptors and may be impacted by many factors such as age, nutrition status, hypoglycemia, disease processes, blood loss, and medication administration. Puerperal causes of hypothermia are numerous, including hypovolemia from hemorrhage and disseminated intravascular coagulopathy, aggressive fluid resuscitation, general or neuraxial anesthesia, as well as administration of medications commonly used in labor and delivery including oxytocin and magnesium sulfate, among others [4-9].

When hypothermia occurs, metabolism, ventilation, and cardiac output are initially increased to maintain bodily function. Clinical presentation depends on the severity of the hypothermia [1]. As hypothermia worsens, organ systems including neurologic, metabolic, and cardiac systems shut down and lead to death. Significant for the postpartum patient, even mild hypothermia (<1 degree C) causes functional impairment of platelets and enzymes within the coagulation cascade. In surgical patients, duration of hypothermia is directly related to the requirement for transfusion of blood products. Rapid evaluation and intervention are crucial [3-5, 10].

In this case of hypothermia following vaginal delivery, many potential etiologies were considered and evaluated. Of these, the most likely etiologies include the following: KCl replacement using chilled fluids, rapid postpartum fluid resuscitation, physiologic vasodilation of pregnancy, and episodic hypothermia with hyperhidrosis.

This patient had several predisposing factors placing her at risk for hypothermia. First, her age: pediatric and adolescent patients lose heat more quickly than adults due to their generally large surface-to-mass ratio and decreased subcutaneous adipocyte tissue insulating layer leading to impaired thermoregulation. Of note, her body mass index (BMI) at the time of delivery was 23.8. Second, her recent emergency childbirth: physiologic dilation of pregnancy, which persists until several weeks postpartum, increases blood flow to the skin and potentiates convective and conductive heat loss. Increased sweating from labor effort may have then caused heat loss through evaporation. Additionally, extreme physical exertion is associated with secondary hypothermia due to insufficient energy for thermoregulation [2,5,11].

Postpartum thermoregulation is also directly impacted by several common and uncommon pathologies associated with pregnancy and delivery. These were considered and ultimately excluded in the case of this patient.

Hemorrhage and resultant hypovolemic shock are associated with hypothermia, and hypothermia, in turn, worsens bleeding by direct effect on platelet function via impaired release of thromboxane A2 and impaired coagulation cascade enzyme function [10,12]. This patient’s total delivery blood loss was not significant and her postpartum lochia was minimal. Her abdomen was nonacute and her hemoglobin remained stable postpartum, therefore concealed internal bleeding was excluded.

Sepsis may also cause hypothermia due to the generalized peripheral vasodilation and loss of endothelial integrity which interferes with thermoregulation. Evaluation for sepsis in this patient included serial complete blood counts, serum lactate, and blood cultures, and she was administered empiric ceftriaxone to treat a possible occult infection. However, her laboratory results remained within normal limits and stable on serial checks. She exhibited no clinical signs or symptoms of endometritis, urinary tract infection, respiratory infection, or thromboembolism. Blood cultures returned negative at 52 hours and antibiotics were subsequently discontinued.

Hypophyseal malfunction may be associated with late pregnancy or the early postpartum period, as the stress of pregnancy and delivery can at times unmask an underlying condition. Lymphocytic adenohypophysitis is one type of hypophyseal disorder that has been uncovered in this fashion and may cause symptoms including hypothermia with presentations similar to sellar compression, diabetes insipidus, hypopituitarism, hypoglycemia, or hyperprolactinemia [13]. Laboratory evaluation and head imaging should be considered to assess for signs of lymphocytic adenohypophysitis. No head imaging was pursued in this case due to the lack of any supporting laboratory abnormalities and the relatively quick resolution of symptoms.

Underlying maternal cardiac disease was considered given her history of childhood heart murmur. This patient’s EKG showed abnormalities during the hypothermic period which were consistent with known effects of hypothermia and resolved with return to normothermia, therefore more likely to be a consequence, rather than a cause, of her hypothermia. Outpatient evaluation with cardiology was recommended, however, the patient did not pursue follow-up.

Additional maternal causes of hypothermia that were considered and ultimately excluded in this patient include hypothyroidism, hypoglycemia, adrenal insufficiency, and episodic spontaneous hypothermia with hyperhidrosis (Shapiro’s syndrome) [3,14,15].

Iatrogenic hypothermia may result from medications commonly administered during labor, delivery, and in the immediate postpartum period. In animal models, oxytocin administration has been associated with hypothermia, however, this patient did not receive supplemental oxytocin during labor or postpartum due to the precipitous nature of her delivery and subsequent light lochia [6]. Several cases of magnesium sulfate administration-related hypothermia have been reported, thought to be related to toxicity and resultant muscular paralysis, respiratory depression, and cardiac compromise [7-9]. In patients undergoing cesarean delivery, perioperative hypothermia commonly occurs in patients who receive general or neuraxial anesthesia due to thermal redistribution to peripheral tissues, impaired vasomotor and shivering responses, and administration of intrathecal opioids [1,3,16]. International standard guidelines recommend accurate patient core temperature monitoring perioperatively, and use of active warming methods including forced air warming, administration of warmed IV fluids, and increased operating room temperature for prevention of hypothermia [1,2,17]. Given the precipitous nature of her delivery, however, this patient did not receive any anesthetic agents.

Iatrogenic hypothermia may also occur in the setting of volume replacement and electrolyte repletion, especially when fluids are not warmed prior to administration. The patient in this case did receive IV fluids, including KCl replacement on two occasions, three hours apart, prior to developing hypothermia. KCl in our facility is refrigerated.

## Conclusions

In this case of postpartum hypothermia following vaginal delivery in a pediatric patient, the etiology is likely multifactorial. Her young age, physiologic vasodilation of pregnancy, and the administration of chilled fluids in the setting of blood loss of delivery are all contributing factors. In all cases of postpartum hypothermia, it is essential to consider both common and unusual causes. Postpartum care teams should perform early and frequent assessment of vital signs, including core temperature. Immediate intervention for hypothermia should include forced air warming system and warmed IV fluid administration using products and quantities to be chosen based on blood loss, electrolyte levels, and dilution, regardless of route of delivery.
